# Vitamin D and Bone Metabolism in Adult Patients with Neurofibromatosis Type 1

**DOI:** 10.3390/metabo13020255

**Published:** 2023-02-09

**Authors:** Roberta Modica, Barbara Altieri, Francesco D’Aniello, Elio Benevento, Giuseppe Cannavale, Roberto Minotta, Alessia Liccardi, Annamaria Colao, Antongiulio Faggiano

**Affiliations:** 1 Endocrinology, Diabetology and Andrology Unit, Department of Clinical Medicine and Surgery, Federico II University of Naples, 80131 Naples, Italy; 2 Division of Endocrinology and Diabetes, Department of Internal Medicine, University Hospital of Würzburg, 97080 Würzburg, Germany; 3 Pediatric University Department, Bambino Gesù Children’s Hospital, University of Rome “Tor Vergata”, 00165 Rome, Italy; 4 UNESCO Chair, Education for Health and Sustainable Development, Federico II University, 80131 Naples, Italy; 5 Endocrinology Unit, Department of Clinical and Molecular Medicine, Sant’Andrea Hospital, ENETS Center of Excellence, Sapienza University of Rome, 00189 Rome, Italy

**Keywords:** neurofibromatosis type 1, vitamin D, bone metabolism, osteoporosis, tumor

## Abstract

Neurofibromatosis type 1 (NF1) is a genetic multisystemic autosomal dominant disorder determining reduced life expectancy due to higher risk of developing benign and malignant tumors. Low levels of vitamin D and reduced bone mineral density (BMD) have been reported in young patients with NF1. However, correlation between vitamin D and NF1 phenotype needs to be elucidated. Aim of this study was to assess vitamin D levels and bone metabolism in NF1 patients, analyzing potential correlations with clinical phenotype. A cross-sectional study was carried out in a monocentric series of NF1 patients, evaluating genotype, clinical phenotype, BMD, biochemical evaluation with focus on serum 25OH-vitamin D, parathyroid hormone (PTH), calcium and phosphate levels. Correlations between clinical manifestations, neurofibromas, and vitamin D status have been studied in comparison with healthy controls. 31 NF1 adult patients were matched for sex, age and body mass index with 31 healthy controls. A significantly difference in vitamin D level emerged in NF1 patients compared to controls. Interestingly low vitamin D levels correlated with a more aggressive phenotype and with a bigger size of neurofibromas. These data underline that vitamin D deficiency/insufficiency may play a role in clinical severity of neurofibromas in patients with NF1, suggesting the need to check bone status and replace vitamin D in these patients.

## 1. Introduction

Neurofibromatosis type 1 (NF1), also known as von Recklinghausen disease, is a genetic multisystemic autosomal dominant disorder. The reported prevalence varies from 1:3500 to 1:4500 people [1,2] and recent studies show an increased incidence of about 1:2000 [3]. The causing mutations affect the NF1 gene, mapped on chromosome 17q11.2, encoding for neurofibromin, a tumor suppressor protein expressed in many cells, involved in cell proliferation and differentiation. Therefore, NF1 patients have a higher risk than general population of developing both benign and less frequently malignant tumors, due to uncontrolled cell proliferation [4,5]. NF1 has relevant inter-familial and intra-familial phenotypic variability and the penetrance is almost complete in adults [6]. NF1 diagnosis is based on criteria established by the National Institutes of Health (NIH) Consensus Development Conference in 1988 requiring at least two among the following: (1) six or more café-au-lait spots (CLS) over 5 mm in diameter in prepubertal individuals and over 15 mm in greatest diameter in post pubertal individuals; (2) two or more neurofibromas of any type or one plexiform neurofibroma; (3) freckling in the axillary or inguinal regions; (4) two or more Lisch nodules (iris hamartomas); (5) optic glioma; (6) a distinctive bone lesion such as sphenoid dysplasia or thinning of long bone cortex, with or without pseudarthrosis; (7) first-degree relative (parent, sibling, or offspring) with NF1 by the above criteria [7]. Importantly NF1 leads to a reduction in life expectancy of 10–15 years, in comparison with general population, mainly in women, due to increased risk of malignancy [8,9]. Furthermore, bone manifestations in NF1 are frequent and may result in significant morbidity [10]. Consequently, the impairment of bone metabolism has emerged as a relevant issue, alongside with the role of vitamin D [11]. Diverse skeletal abnormalities, including scoliosis, are commonly described [10] and NF1 patients have a higher prevalence of osteoporosis and osteopenia than general population [12,13,14]. The pathogenesis of bone diseases is complex and not completely understood, although alterations in functions and proliferation of osteoclasts and osteoblasts have been reported in mouse models [15,16]. A key role in bone metabolism is played by 25-hydroxy-vitamin D (25OHD) as it regulates the dietary absorption of calcium and phosphorus, although several “non calcemic” effects have been proposed [17,18,19].

Average concentrations of 25OHD have been proven to be significantly lower in NF1 patients, with a prevalence of hypovitaminosis ranging from 60% to 98% [12,14,17,20]. Noteworthy, vitamin D replacement therapy seems to decrease the bone demineralization in patients maintaining 25OHD levels above 30 ng/dL [17,21]. The mechanisms beneath the correlation between vitamin D and NF1 phenotype still need to be elucidated.

Serum 25OHD levels have been recently reported to be inversely correlated with the number of dermal neurofibromas. On the contrary, this association was not described with plexiform neurofibromas and malignant peripheral nerve sheath tumors [22].

An increased knowledge of the pathogenesis of bone alterations, as well as other clinical manifestation in NF1 is needed to improve prevention and follow up strategies in these patients. Therefore, this study aimed out to evaluate vitamin D levels and bone metabolism in NF1 adult patients, providing possible relationship with clinical manifestations of the syndrome.

## 2. Experimental Design

### 2.1. Design and Setting

This is a retrospective cross-sectional monocentric study including patients with NF1 matched 1:1 with healthy controls by sex, age and body mass index (BMI). Controls were selected from healthy volunteers from the hospital and employees and coming from the same geographical area of the patients. The patients were diagnosed with NF1 according to NIH criteria [6]. Differential diagnosis with Legius or other genetic syndromes has been taken into consideration with both clinical features and genetic analysis when available. Except for 9 NF1 patients (29%) who were inadequately supplemented with vitamin D (less than 600 IU daily) [23], all participants were vitamin D treatment naïve or were not treated with drugs known to interfere with bone and mineral metabolism. The study was carried out at the Endocrinology Unit of the Federico II University of Naples, Italy. Patients’ clinical and biochemical data were obtained from both clinical assessment and medical records from 2010. Last follow-up was December 2021. The study was conducted in accordance with the Declaration of Helsinki and approved by the Ethical Committee of the “Federico II” University of Naples (n. 201/17). All subjects signed an informed consent form.

### 2.2. Clinical Assessment

Anthropometric measurements, clinical, biochemical, and imaging assessment of both patients and controls, as well as the disease status for NF1 patients, were evaluated at the time of first access. Clinical assessment included sex, age, BMI for both patients and controls, and evaluation of the 7 main clinical features according to NIH criteria for NF1 patients. BMI was calculated by weight and height (weight (kg) divided by height squared (m^2^), kg/m^2^] following standard criteria [24,25]. According to WHO’s criteria, patients were classified by BMI as normal weight (BMI 18.5–24.9 kg/m^2^), overweight (BMI 25.0–29.9 kg/m^2^), and obese (BMI ≥ 30.0 kg/m^2^ [24]. In NF1 patients, number, and size of neurofibromas were evaluated examining the entire body. An eye examination with slit lamp was performed to assess Lisch nodules. Brain contrast-enhanced MRI evaluated optic glioma. Two ad hoc scores were arbitrarily designed to assess the extent of clinical manifestations with the aim to ease patients’ analysis: N-Score, to evaluate the level of burden caused by the presence of neurofibromas, and the Severity Index Score (SIS) to evaluate clinical diagnostic criteria of the syndrome. The N-score was scored from 0 to 3 and consisted of three parameters: (1) distribution of neurofibromas (localized = 0, spread = 1), (2) size of neurofibromas (<5 cm = 0, ≥5 cm = 1), (3) surgical removal of neurofibromas (no = 0, yes = 1). Surgical removal of neurofibroma was included as part of the severity score as it reflects either a need of the patient for discomfort or cosmetic disfigurement or even a clinical suggestion. According to N-score the patients were divided into two groups: N-score = 0–1 (low burden) and N-score = 2–3 (high burden). The SIS was from 0 to 6, and evaluated clinical diagnostic criteria of the syndrome, except the familiarity for NF1 because not directly related with clinical severity. The presence of each criterion was scored with 1, whilst its absence corresponded to 0. According to the SIS score, the patients were divided into three groups: SIS = 1–2 (low); SIS = 3–4 (medium); SIS = 5–6 (high). We decided to arbitrarily design these two scores due to the lack of validated evaluation scores for NF1, despite some limitations including the subjective choice of parameters and the small sample size that may have an impact on results.

### 2.3. Biochemical Assessment

Blood samples for biochemical assessment were obtained in the morning, after 8 h-fasting. Serum calcium, phosphorus and albumin were measured by automatic technique (Roche Modular System, Basel, Switzerland). Albumin-corrected serum calcium was calculated using the following formula: corrected Ca (mg/dL) = measured total Ca (mg/dL) + 0.8 × –4.0 − serum albumin [g/dL]), with 4.0 representing the average albumin and 0.8 the correction factor [26]. Intact parathyroid hormone (PTH) was evaluated by immunometric assay (Immulite iPTH from Diagnostics Products Corporation, Los Angeles, CA, USA). 25OHD levels were quantified by a competitive chemiluminescence immunoassay (DiaSorin Liaison, Saluggia, Italy) [26]. All participants were classified in 4 different categories according to 25OHD levels defined by the Endocrine Society Guidelines [23]: severe deficiency (<10 ng/mL); deficiency (≥10 ng/mL; <20 ng/mL); insufficiency (≥20 ng/mL; <30 ng/mL); sufficiency (≥30 ng/mL). A comparative analysis of serum 25OHD levels, albumin-corrected serum calcium, phosphorus and PTH levels between NF1patients and controls was carried out.

### 2.4. Genetic Test for Germline NF1 Variants

Confirmatory genetic testing for germline NF1 variants was performed in 14 patients. DNA was isolated from peripherical blood with MagCore Genomic DNA Whole Blood Kit (RBC Bioscience Corp., Taiwan) according to manufacturer’s instructions. For 10 patients, libraries were prepared with SeqCap EZ HyperCAp Library (Roche, IN, USA) and sequenced on an Illumina NovaSeq 6000 (San Diego, CA, USA) platform and 150-bp paired end reads. Raw data were aligned and analyzed with Burrows–Wheeler Aligner tool. In 4 patients, NF1 variations were genotype by Polymerase Chain Reaction (PCR) according to Sanger’s method. To test whether newfound NF1 variants might be causative of the pathology, the prediction program Mutation Taster was used.

### 2.5. Bone Evaluation

Bone Mineral Density (BMD) obtained with Computerized Bone Mineralometry with Dual X-ray Absorptiometry (CBM-DXA) was available in 24 patients with NF1. Osteopenia or osteoporosis was classified according to the WHO criteria: osteopenia defined as BMD between 1.0 and 2.5 Standard Deviation (SD) (T-score between −1.0 and −2.5), and with osteoporosis defined as a BMD ≤ 2.5 SD (T score ≤ −2.5) [27,28]. Deviations of spine were studied through X-ray of dorso-lumbar tract obtained in 17 patients.

### 2.6. Statistical Analysis

Statistical analysis was carried out with SPSS version 29.0 software for Windows (SPSS Inc., Chicago, IL, USA) and GraphPad Prism (version 9.0, La Jolla, CA, USA). The data are expressed as mean ± SD for numeric variables and as percentages for categorical variables. Data distribution was assessed by the Shapiro–Wilk test. Non-parametric Mann-Whitney test and Chi-Square test were used to compare continuous or dichotomic variables, respectively. *p*-value < 0.05 was considered as statistically significant. Sensitivity analyses have not been performed due to the relatively small sample size.

## 3. Results

We included 31 patients with NF1 (21 women and 10 men, mean age 41 ± 11.4 years, range 23–67 years), matched for age, sex and BMI with 31 healthy controls. Clinical characteristics and levels of parameters involved in bone metabolism in NF1 patients and controls are summarized in Table 1.

Mean age at NF1 diagnosis was 18.6 ± 13.2 years, whereas mean age at the time of the study was 41 ± 11.4. The 83.9% of patients (*n* = 26) were shorter than mean value of height reported in Italy [29]. More specifically, 8 (22.6%) patients resulted below 2 SD from the average height of the country and 58% were overweight or obese. In this case, 15 NF1 patients had a degree of kinship (7 families). Among the subgroup of patients evaluated by confirmatory genetic test, we found 2 new NF1 variants: c.430delT (p.Ser144LeufsTer21) and c.4430 + 2T > A. Particularly, the NF1 variant c.430delT (p.Ser144LeufsTer21) could lead to an addition stop codon. However, this variant has not been subjected to a functional study yet. The NF1 variant c.4430 + 2T > A resulted as probably pathogenetic.

### 3.1. Diagnostic Criteria of NF1

Clinical signs and symptoms of subjects are summarized in Table 2. CLS were detected in 28 (90.3%) subjects and were mainly located in abdomen and/or thorax. Neurofibromas, were diagnosed in 29 (93.5%) patients. Abdomen, chest and limbs were the most common localizations of neurofibromas. In this case, 12 patients (38.7%) underwent surgery to remove neurofibromas, both for functional and/or aesthetic reasons. Plexiform neurofibromas were identified in 3 subjects (9.7%). Axillary and inguinal freckling were found in 13 (41.9%) patients. The presence of Lisch nodules was demonstrated in 17 (54.8%) patients. Optic pathway glioma was observed in 7 (22.6%) patients, with one case of bilateral localization. Two of them were treated, respectively, one with surgery and one with radiotherapy. The surgically treated patient suffered from GH deficiency and hypothyroidism after surgery and the patient treated with radiotherapy during childhood developed hypopituitarism associated to empty-sella and lately developed secondary glioblastoma multiforme [30,31]. Regarding bone, 17 subjects (54.8%) presented signs associated with NF1. In this case, 13 patients (41.9%) presented scoliosis, but no case of dystrophic scoliosis was detected. Among the included patients, 25 subjects (80.6%) had a first-degree relative affected, but for 10 of them the relative was not followed by the center of the study. N-score was low in most of patients (19 out 31, 61.3%). Looking further into the parameters of the N-score: 19 patients (61.3%) presented a widespread distribution of neurofibromas; 12 subjects (38.7%) had at least one neurofibroma bigger than 5 cm; 12 subjects (38.7%) resorted to surgery. SIS was scored low in 7 (22.6%) patients, medium in 14 (45.2%) patients and high in 10 (32.2%), as detailed in Table 3.

### 3.2. Other NF1 Clinical Manifestations

Tumors, either benign or malignant, were detected in 11 (35.5%) patients. Brain was the most involved organ, with 3 patients reporting a central nervous system tumor, including 1 cerebellar astrocytoma, 1 low grade protoplasmic microcystic astrocytoma and pituitary adenoma, and 1 glioblastoma multiforme secondary to radiotherapy. Other tumors were: 2 uterine myomas; 2 pheochromocytomas; multiple osteofibromas in 1 patient; 1 metastatic colon carcinoma; 1 medullary thyroid carcinoma; 1 Hodgkin lymphoma. Noteworthy, we did not diagnose any malignant peripheral nerve sheath tumor. Frequent and repeated episodes of headache of variable intensity were reported by 21 (67.7%) patients.

### 3.3. 25OHD and Bone Metabolism

**Figure 1:**
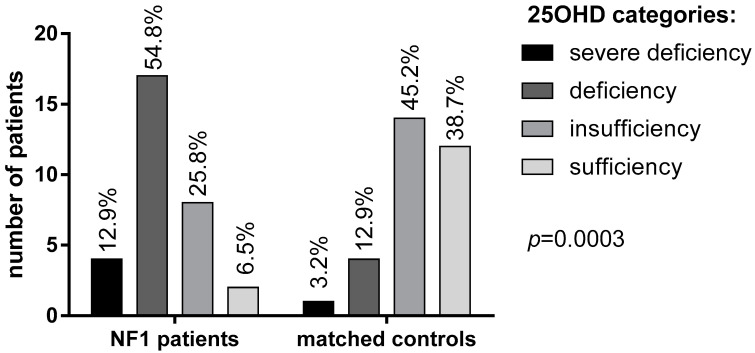
25OHD levels in patients with NF1 and controls according to the Endocrine Society Guidelines [23].

Mean 25OHD levels in NF1 patients were significantly lower than in the control group (16.4 ± 6 vs. 27.2 ± 9.2 ng/mL, respectively, Table 1). Specifically, 25OHD deficiency and severe deficiency was found in a significantly larger proportion of NF1 patients (54.8% and 12.9%, respectively) in comparison with healthy controls (12.9% and 3.2%, respectively; *p* = 0.0003, Figure 1).

**Figure 2:**
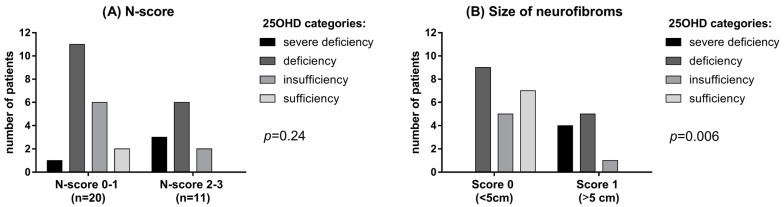
(**A**) 25OHD level in patients with different N-score. (**B**) 25OHD in patients with neurofibromas of different dimension.

Only 2 NF1 patients (6.5%) had sufficient levels (>30 ng/mL) of vitamin D compared to 12 subjects (38.7%) in the control group. Mean albumin-corrected serum calcium and phosphorus concentrations were within the normal range in both patients and controls, although phosphorus was significantly lower in NF1 patients than controls (*p* = 0.035, Table 1). No significant difference was observed in PTH levels, although a trend of higher PTH levels was found in NF1 patients (42.3 ± 31.9 vs. 23.8 ± 15.5 pg/mL, Table 1). A possible correlation between vitamin D deficiency and neurofibromas has been investigated using the N-score. In patients with a higher N-score (score 2–3) lower vitamin D levels were detected, though not significantly, than in patients with lower N-score (score 0–1) (*p* = 0.24; Figure 2B). Interestingly, a large size (>5 cm) of neurofibromas was significantly related with 25OHD deficiency (*p* = 0.006; Figure 2B).

On the other hand, the distribution and the need for surgical removal did not significantly correlate with 25OHD levels (respectively, *p* = 0.19 and *p* = 0.59). Furthermore, 25OHD levels did not correlate with SIS (*p* = 0.72), presence of glioma (*p* = 0.36), presence of specific bone lesions (*p* = 0.26), and occurrence of other tumors (*p* = 0.18). MOC-DEXA assessment regarding femoral data showed: osteopenia in 13 out of 24 (54.2%) and osteoporosis in 1 out of 24 (4.2%). Regarding lumbar spine data, osteopenia was present in 9 out of 24 (37.5%) and osteoporosis in 3 out of 24 (12.5%). These data show an overall higher rate of osteoporosis and osteopenia in NF1 patients than in general populations [13]. In detail the 3 cases of lumbar osteoporosis were detected in patients younger than 50 years of age and the youngest was a 36-year-old male.

## 4. Discussion

This study provides an assessment of vitamin D levels and bone metabolism in a monocentric cohort of adult NF1 patients, analyzing potential correlations with clinical phenotype. Low levels of 25OHD in individuals with NF1 had already been reported [12,14,17,20] and treatment with vitamin D have been proven to be effective in improving DEXA data in NF1 young patients, including children [11]. Our results confirm and strengthen the evidence of a very high prevalence of low 25OHD levels in adult NF1 patients. 25OHD deficiency was found in 17 (54.8%) patients against 4 (12.9%) in the control group, while a status of severe deficiency was detected in 4 (12.9%) NF1 patients, against 1 control (3.2%). To note, controls were matched not only for age and sex but also for BMI, reducing potential bias in the evaluation of 25OHD. Low 25OHD levels recorded in 93.5% (29 out of 31) patients are higher compared to previous observations (56%—Tucker et al. (2009) [32]; ~60% Petramala et al. (2012) [14]), but consistent with more recent findings (84%—Jalabert et al. (2021) [12]; 98.6%—Filopanti et al. (2019) [20]). Literature data regarding potential correlation between 25OHD levels and genotype are lacking, but it could be of interest to deepen this relationship in light of our findings.

Conflicting data exists regarding calcium-phosphate metabolism in NF1 [11,20,33]. We did not find any statistically significant difference between patients and controls regarding the relationship between corrected calcium, PTH levels and low 25OHD. Particularly, phosphoremia levels were lower in NF1 patients, though within the normal range. The lower phosphoremia could be related to the lower vitamin D and higher PTH levels observed in NF1 patients [25]. However, the clinical meaning of this finding is unclear and has to be confirmed and explained in further studies. Our attempt to identify a correlation between clinical severity of NF1 and 25OHD levels, take advantage of the N-score. Noteworthy, an association between the dimension of neurofibromas and low 25OHD levels has been detected. To our knowledge, this is the first time that this evidence is described in literature, while an inverse relationship between 25OHD levels and number of neurofibromas has been already described [22,34]. Interestingly, the latter correlation was not confirmed by our data. However, this contrasting result could be related to the smaller cohort of patients included in our study. No statistically significant difference was found instead between 25OHD levels and SIS score. The instrumental evaluation with MOC-DEXA highlighted osteopenia, femoral and lumbar in 54.2% and 37.5% of cases, respectively, and osteoporosis, femoral and lumbar in 4.2% and 12.5% of cases, respectively. Previous studies supported the evidence that higher bone demineralization is more frequent in NF1 subjects than in the general population [13,14,17,20,32]. In these studies, the prevalence of osteopenia and osteoporosis ranged 44–50% and 18–19%, respectively [14,32]. The results obtained about other bone alterations are consistent with previous literature. More specifically, the prevalence of scoliosis was 41.9% in our population against the 30% reported by Filopanti et al. (2019) [20] and Gutmann et al. (2017) [35]. Tibial pseudoarthrosis was observed in 9.7% of cases, similar to Jalabert et al. (2021) [12]. Moreover, sphenoid dysplasia was described in literature with a variable prevalence from 3% to 11% [36]. We had 1 patient (3.2%) with sphenoid dysplasia. Data about height are conflicting in NF1 patients, but a role of 25OHD cannot be excluded. In this study 83.9% of patients were shorter than mean value of height reported in Italy [29]. To note, the prevalence of short stature in our study was 22.6% (8/31 patients) against 8% reported by Soucy et al. (2013) [37] and, more recently, 28.3% reported by Souza et al. (2016) [38]. The most prevalent clinical manifestations in our population were neurofibromas (93.5%) and CLS (90.3%). These clinical features are also reported among the most frequent in recent literature [39], but with a predominance of CLS (96.5%) and an inferior rate of neurofibromas (78.1%). This last discrepancy may be due to our small sample size. A plexiform neurofibroma was detected in 3 subjects (9.7%). This finding could be an underestimation because it’s usually difficult to clinically diagnose these lesions, which often remain undiagnosed [40,41]. The percentage of patient with axillary and inguinal freckling was considerably lower than reported by other authors (41.9% vs. 90%) [39]. Lisch nodules presence was lower compared to past studies (54.8% vs. 78%) [42]. Instead, the percentage of optic glioma was similar (22.6% vs. 15–20%) [43]. In addition to neurofibromas, other tumors were detected in our population. Here, 11 (35.5%) patients had a tumor different from neurofibromas and optic pathway gliomas. Brain was the mainly involved organ with a percentage of 9.7% vs. 1–5% highlighted in literature [44,45]. Interestingly, 25OHD does not correlate with any other clinical manifestation of the syndrome and is not associated with tumor onset. Several limitations should be considered when interpreting the results of this study including mainly the retrospective design with all the inherent biases of this methodology, and the arbitrarily designed scores to assess the extent of clinical manifestations. We acknowledge that seasonal variations of vitamin D levels dosage may occur impacting results and our study may inaccurately represent the general NF1 population for small sample size and heterogeneity of the disease. Furthermore, sensitivity analyses and comparison between gender or other subgroups have not been performed due to the small sample size. Nevertheless, our results support the need to increase our knowledge regarding the role of vitamin D in NF1 patients and its role in bone metabolism.

## 5. Conclusions

This study highlights that vitamin D may play a significative role in clinical manifestations of NF1 and BMD evaluation is a relevant issue in these patients, despite the relatively small sample size. Two new NF1 mutations have been identified, but correlation between genotype and phenotype is lacking. Here, we provide further validation to the finding of vitamin D deficiency/insufficiency in NF1 and suggest further studies to both prospectively confirm the correlation between vitamin D and neurofibromas and evaluate the potential role of vitamin D supplementation in an interventional trial.

## Figures and Tables

**Table 1:** Clinical characteristics and parameters involved in bone metabolism in patients with NF1 and matched controls.

Parameters	NF1 Patients	Controls	*p*-Value
Age at time of the study, years	41 ± 11.4	43.8 ± 7.2	0.106
BMI, kg/m^2^	27 ± 5.8	25.5 ± 4.2	0.406
25OHD, ng/mL	16.4 ± 6	27.2 ± 9.2	<0.0001
Albumin-corrected serum calcium, mg/dL	9.4 ± 0.4	9.3 ± 0.4	0.423
Phosphorus, mg/dL	3.6 ± 0.5	3.9 ± 0.5	0.035
PTH, pg/mL	42.3 ± 31.9	23.8 ± 15.5	0.077

Values are reported as mean ± standard deviation. Abbreviations: BMI, body mass index; PTH, parathyroid hormone; 25OHD, 25-hydroxy-vitamin D.

**Table 2:** Clinical signs of affected population.

Clinical Signs	Patients (n.)
Café-au-lait spots (CLS)	28 (90.3%)
Neurofibromas	29 (93.5%)
Surgically treated neurofibromas	12 (38.7%)
Plexiform neurofibromas	3 (9.7%)
Freckling	13 (41.9%)
Lisch nodules	17 (54.8%)
Optic pathway glioma	7 (22.6%)
Bone signs	17 (54.8%)
Scoliosis	13 (41.9%)
Tibial pseudoarthrosis	3 (9.7%)
Sphenoidal dysplasia	1 (3.2%)

**Table 3:** Severity index score (SIS) score for each patient.

Patients ID	CLS ≥ 6	Neurofibromas ≥ 2 or 1 Plexiform	Axillary or Inguinal Freckling	Optic Glioma	Lisch Nodule	Osseous Lesions	SIS Score
1	1	1	0	1	1	0	4
2	1	1	1	0	0	0	3
3	0	1	0	0	0	0	1
4	1	1	1	0	1	1	5
5	1	0	1	0	0	0	2
6	1	1	1	0	1	1	5
7	1	1	0	0	1	1	4
8	0	1	0	0	0	0	1
9	1	1	1	0	1	1	5
10	1	1	0	0	0	1	3
11	0	1	0	0	1	1	3
12	1	1	1	0	1	0	4
13	1	1	0	1	1	1	5
14	1	1	0	1	0	0	3
15	1	1	1	0	1	1	5
16	1	1	1	1	0	0	4
17	1	1	0	0	1	0	3
18	1	1	0	0	1	0	3
19	1	1	0	0	0	0	2
20	1	1	0	0	0	0	2
21	1	1	1	1	0	0	4
22	1	1	0	0	0	0	2
23	1	1	1	0	1	1	5
24	1	1	1	0	1	1	5
25	1	1	1	0	1	1	5
26	1	1	1	0	0	1	4
27	1	1	0	0	0	1	3
28	1	1	0	1	1	1	5
29	1	1	0	0	1	1	4
30	1	1	0	1	1	1	5
31	1	0	0	0	0	0	1

Values are reported as 0 stands for “criteria is not respected” and 1 stands for “criteria is respected”. Abbreviation: CLS, Cafè au-Lait Spots; SIS, Severity Index Score.

## Data Availability

The data presented in this study are available on request from the corresponding author. The data are not publicly available due to privacy.
